# Circulating Chromogranin A as A Marker for Monitoring Clinical Response in Advanced Gastroenteropancreatic Neuroendocrine Tumors

**DOI:** 10.1371/journal.pone.0154679

**Published:** 2016-05-09

**Authors:** Tiantian Tian, Jing Gao, Na Li, Yanyan Li, Ming Lu, Zhongwu Li, Zhihao Lu, Jie Li, Lin Shen

**Affiliations:** 1 Department of Gastrointestinal Oncology, Key laboratory of Carcinogenesis and Translational Research (Ministry of Education), Peking University Cancer Hospital & Institute, Beijing, China; 2 Department of Pathology, Key laboratory of Carcinogenesis and Translational Research (Ministry of Education), Peking University Cancer Hospital & Institute, Beijing, China; UPR 3212 CNRS -Université de Strasbourg, FRANCE

## Abstract

Chromogranin A (CgA), present in the chromaffin granules of neuroendocrine cells, is a useful biomarker for the diagnosis of patients with gastroenteropancreatic neuroendocrine tumors (GEP-NETs). This study was conducted to investigate the potential role of circulating CgA in monitoring clinical response in Chinese patients with advanced GEP-NETs. Eighty patients with advanced GEP-NETs treated in Peking University Cancer Hospital from September 2011 to May 2014 and 65 healthy individuals were included in this study. Serum CgA levels were analyzed for relationship with patient’s baseline characteristics and clinical outcome. Median CgA levels were significantly higher in patients with advanced GEP-NETs than in healthy individuals (93.8 ng/mL vs. 37.1 ng/mL; P<0.01), as well as significantly higher in patients with carcinoid syndrome or liver metastasis than in those without carcinoid syndrome (298.8 ng/mL vs. 82.9 ng/mL; P = 0.011) or liver metastasis (137.0 ng/mL vs. 64.4 ng/mL; P = 0.023). A CgA cutoff value of 46.2 ng/mL was used in this study with a sensitivity of 78.8% and specificity of 73.8%. Patients with CgA levels higher than 46.2 ng/mL had a worse prognosis than patients with CgA levels lower than 46.2 ng/mL (P = 0.045). Notably, a weak correlation was observed between changes in serum CgA levels and clinical response to the IP regimen as well as SSAs. Our data also indicate that serum CgA could be a useful indicator of patient prognosis though there is more research required in order to validate such claims.

## Introduction

Neuroendocrine tumors (NETs), divided into well-differentiated (grade 1 and 2) or poorly differentiated (grade 3) according to WHO 2010 grading classification[1], arise from cells throughout the diffuse endocrine system and comprise a broad family of tumors. The most common type of NETs, gastroenteropancreatic neuroendocrine tumors (GEP-NETs), is the second most prevalent tumors of gastrointestinal tract and its incidence has risen dramatically in the last twenty years [2]. Confounding their treatment and diagnosis, GEP-NETs typically exhibit variable and nonspecific clinical symptoms and most patients have advanced disease (local advanced or metastatic disease) at diagnosis [3]. Histopathology has been the gold standard for diagnosing GEP-NETs though several non-invasive biomarkers discovered in recent years could potentially aid diagnosis. Specifically, peripheral blood containing chromogranin A (CgA) has been reported to be supportive of the clinical diagnosis of GEP-NETs[4, 5].

CgA is an acidic glycoprotein which is specifically expressed in neuroendocrine cells [6]. Despite initial indications of its efficacy as a potent biomarker, a standardized reference value for CgA levels is unavailable due to heterogeneity within patient populations and test methods [7, 8]. Furthermore, a majority of studies which addressed circulating CgA in GEP-NETs patients were conducted utilizing western populations and has been rarely reported in Chinese patients [9].

A safe, economical and convenient method was needed in order to accurately monitor patient response to therapy as an auxiliary method to morphological evaluation utilizing conventional imaging. In addition to its potential diagnostic role, serum CgA has been associated with monitoring clinical response for NETs patients [10–12]. However, due to different types of NETs and different medical treatment, mixed results have been published. For instance, Nehar *et al*. reported that in patients with GEP-NETs and multiple endocrine neoplasia (MEN) who were treated mainly with somatostatin analogs (SSAs), CgA levels and tumor size were closely associated with each other in up to 80% of cases [10]. While in a study of GEP-NETs patients treated with multiple therapies, the concordance between CgA level variations and clinical response was low [13]. Treatment for advanced GEP-NETs comprises palliative resection, SSAs, hepatic-directed therapies, targeted drug, systemic chemotherapy and so on. Currently, there is no standard systemic chemotherapy for GEP-NETs though irinotecan plus cisplatin regimen (IP) has been found to be moderately effective and well tolerated in patients with GEP-NETs in our previous study [14]. Finding the association between serum CgA levels and clinical response and whether serum CgA is useful in evaluating clinical response in patients with advanced GEP-NETs is of most interest to us.

This study was conducted to investigate serum CgA levels in Chinese patients with advanced GEP-NETs and explore its potential role in monitoring clinical response as well as prognosis.

## Materials and Methods

### Patients

Eighty patients with histopathologically confirmed GEP-NETs treated in Peking University Cancer Hospital from September 2011 to May 2014 were retrospectively enrolled in this study. Serum samples from 65 healthy individuals during the same period were used as controls. All patients gave their written informed consents for their blood to be used for research. All clinical data of patients were collected from medical records and the last follow-up was October 2014. Patient records/information was anonymized and de-identified prior to analysis. All the authors had access to identifying information during or after data collection. This study was approved by the medical ethics committee of Peking University Cancer Hospital.

### Treatment regimen and evaluation of clinical outcome

Of the eighty patients, three patients underwent palliative surgery, and seventy-seven patients were administrated with systemic treatment: forty-two patients received IP regimen; twenty-two patients received SSAs and the remaining patients received other treatments such as capecitabine plus oxaliplatin (XELOX) regimen and etoposide. IP regimen chemotherapy was given every two weeks for six cycles or until disease progression, with SSAs as maintenance treatment or other therapies such as hepatic-directed therapy or salvage chemotherapy. Patients treated with SSAs were treated every 4 weeks. All patients were evaluated for clinical response by CT scans every three cycles of therapy. Clinical response was defined as complete response (CR), partial response (PR), stable disease (SD), and progressive disease (PD) according to the Response Evaluation Criteria in Solid Tumors (RECIST) criteria, version 1.1[15]. The overall survival (OS) was calculated from the date of diagnosis to death from any cause.

### Detection of serum CgA

Peripheral blood samples were collected from all participants and control individuals, and centrifuged (2500g) at 4°C for 10 minutes after blood clotting to obtain serum. Samples were then aliquoted and stored at -80°C for further analysis. Serum CgA level was detected using an enzyme-linked immunosorbent assay (ELISA) kit (Chromoa^TM^, Cisbio) according to the manufacturer’s instructions. All patients involved in this study had serum drawn before therapy while forty-nine had serum drawn before and after treatment. All serum samples were measured in triplicate. CgA level changes were considered elevated if there was a ≥25% increase, decreased if there was a ≥25% reduction and stable if there was a <25% increase or a <25% reduction in serum CgA levels[4].

### Detection of other biomarkers for GEP-NETs

Serum NSE (neuron specific enolase), CEA (carcinoembryonic antigen), and CA19-9 (carbohydrate antigen 19–9) were measured according to the instruction of the commercial test Elecsys® NSE (Roche Diagnostics GmbH, Catalogue Number: 12133113122), Elecsys® CEA (Roche Diagnostics GmbH, Catalogue Number: 11731645322) and Elecsys® CA19-9 (Roche Diagnostics GmbH, Catalogue Number: 12133113122). The assays for each marker are designed as sandwich assay based on the Streptavidin-Biotin-technology and they were measured quantitatively on a cobas® e601 platform (Roche Diagnostics GmbH). The cut-off value for NSE (15.2 ng/mL), CEA (5 ng/mL) and CA19-9 (37 U/mL) were determined based on the reference value used in clinical practice.

Immunohistochemical analysis for tissue CgA, Synaptophysin (Syn) and CD56 was performed on a 4 μm thick paraffin-embedded tissue sections according to standardized procedure. The primary antibody used was rabbit anti-Chromogranin A monoclonal antibody (ZA-0507, ZSGB-BIO, Beijing, China), rabbit anti- Synaptophysin monoclonal antibody (ZA-0506, ZSGB-BIO, Beijing, China) and mouse anti-CD56 monoclonal antibody (ZM-0057, ZSGB-BIO, Beijing, China). The results were graded from 0 to 3 according to intensity of staining (0, negative or trace; 1, weak; 2, moderate; 3, intense). Staining of ≥ 1 was considered positive.

### Statistical analysis

Statistical analysis was performed using SPSS 16.0 software. Mann–Whitney test was used to analyze difference of serum CgA levels between patients with GEP-NETs and healthy individuals as well as to compare the levels of serum CgA between groups of patients with different clinical characteristics. Receiver-operating characteristic (ROC) curve was constructed to determine cutoff value of CgA level with the optimal sensitivity and specificity. The correlation between CgA level changes and clinical response were assessed using Spearman correlation test. Kaplan-Meier curve and the log-rank test were used to analyze the correlation of CgA level to survival. The McNemar’s test was used to compare serum CgA to other blood markers. The phi-coefficient correlation test was performed to determine the relationship of CgA expression between tumor tissues and sera. P<0.05 was considered significantly different.

## Results

### Patient characteristics

**Fig 1:**
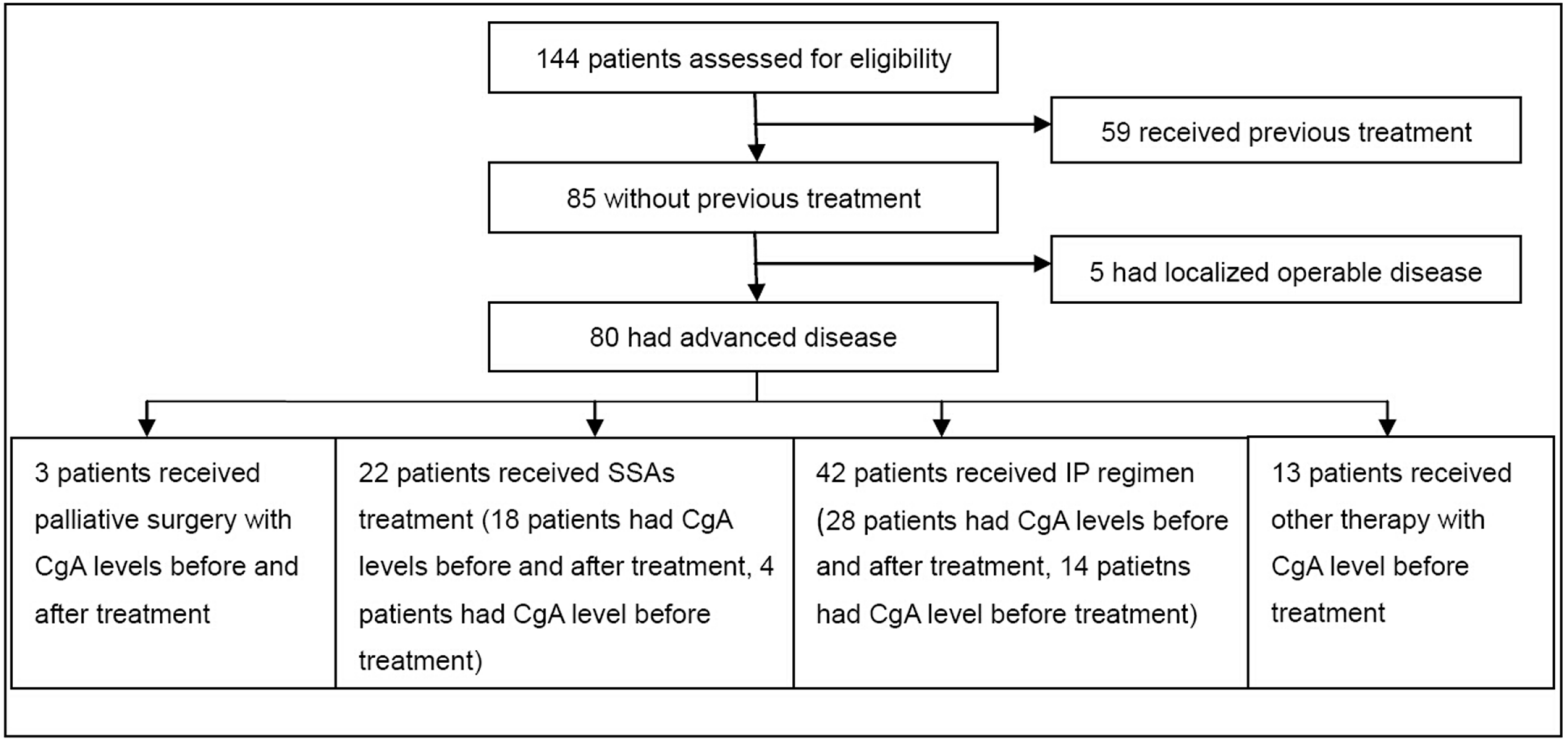
The patient screening process. IP: irinotecan plus cisplatin; SSAs: somatostatin analogs.

Of the eighty patients included in this study fifty were male (62.5%) and thirty were female (37.5%), with a median age of 57.5 years (28–77 years); thirty-nine males (60%) and twenty-six females (40%) with a median age of 50 years (22–75 years) made up the control group. The detailed patients screening process and clinicopathological characteristics of patients were shown in Fig 1 and Table 1. All patients had either locally advanced and unresectable tumors or metastatic tumors (stage III/IV, according to AJCC 2010 staging system[16]) at the time of diagnosis. Histopathological features of patients were shown in S1 Table.

**Table 1:** Clinical characteristics and serum CgA levels of patients.

Characteristics	No. of patients (%)	Median CgA level (ng/mL)	P value*
**Gender**			0.804
Female	30 (37.5%)	121.3 (27.5–1115.8)	
Male	50 (62.5%)	85.7 (21.7–2795.1)	
**Age**			0.674
≤57	40 (50.0%)	143.65 (25.2–1582.6)	
>57	40 (50.0%)	79.8 (21.7–2795.1)	
**Primary sites**			0.197
Esophagus-stomach	34 (42.5%)	89.95 (21.7–2795.1)	
Pancreas	19 (23.8%)	137. 0 (40.8–1582.6)	
Others^#^	27 (33.7%)	73.3 (25.2–913.4)	
**Stage**			0.225
III	13 (16.3%)	56.1 (30.0–455.3)	
IV	67 (83.7%)	80.5 (21.7–2795.1)	
**Grade**			0.165
G1	5 (6.3%)	870.0 (51.9–1115.8)	
G2	22(27.5%)	57.8 (25.2–913.4)	
G3	53 (66.2%)	97.0 (21.7–2795.1)	
**Carcinoid syndrome**			0.011
No	71 (88.8%)	82.9 (21.7–2795.1)	
Yes	9 (11.3%)	298.8 (52.0–913.4)	
**Liver metastasis**			0.023
No	27 (33.8%)	64.4 (27.5–870.0)	
Yes	53 (66.2%)	137.0 (21.7–2795.1)	

^#^Others include colon, duodenum, and unknown primary sites.

*The Mann-Whitney test was used.

### Serum CgA levels in patients and healthy individuals

**Fig 2:**
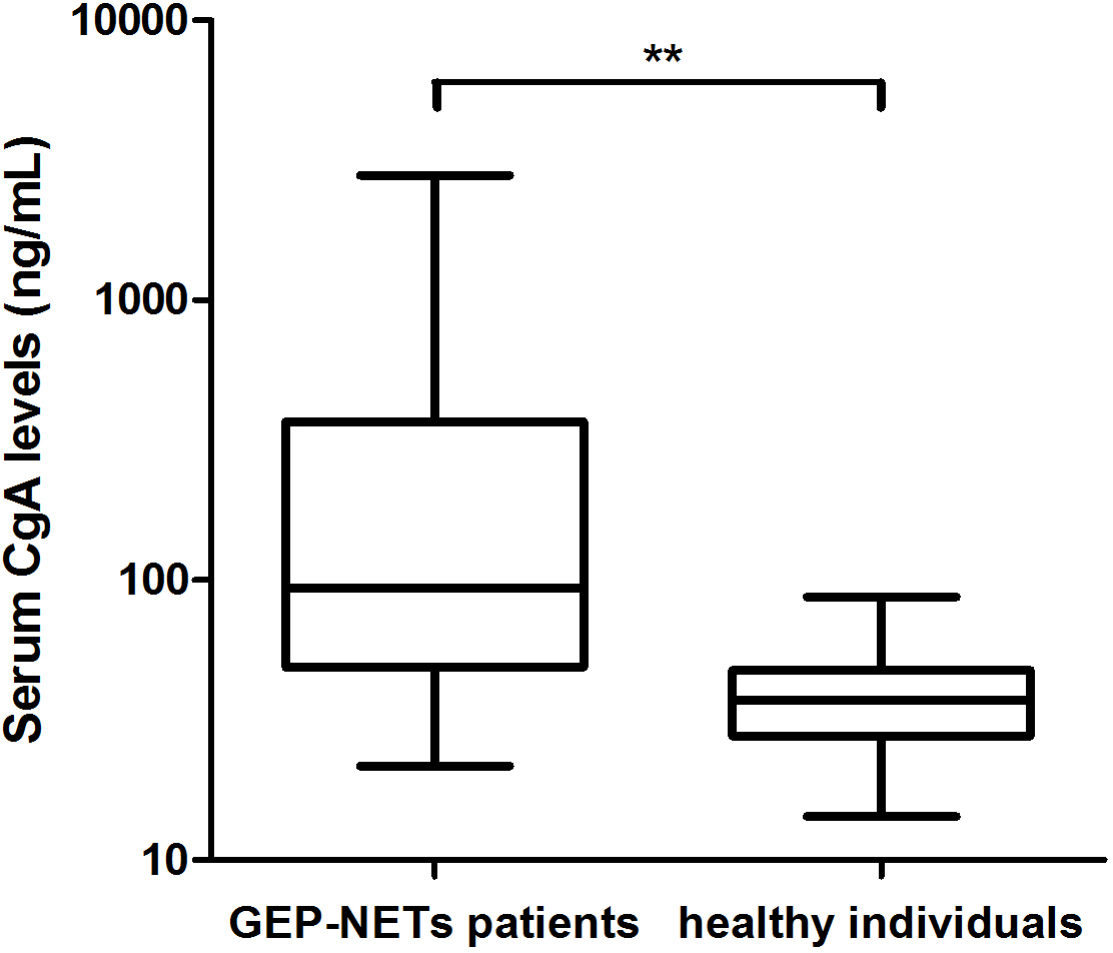
Serum CgA levels in patients with GEP-NETs and healthy individuals. There were higher CgA levels in patients with GEP-NETs compared with healthy individuals (Log10 scale). **P<0.01 (Mann–Whitney test).

**Fig 3:**
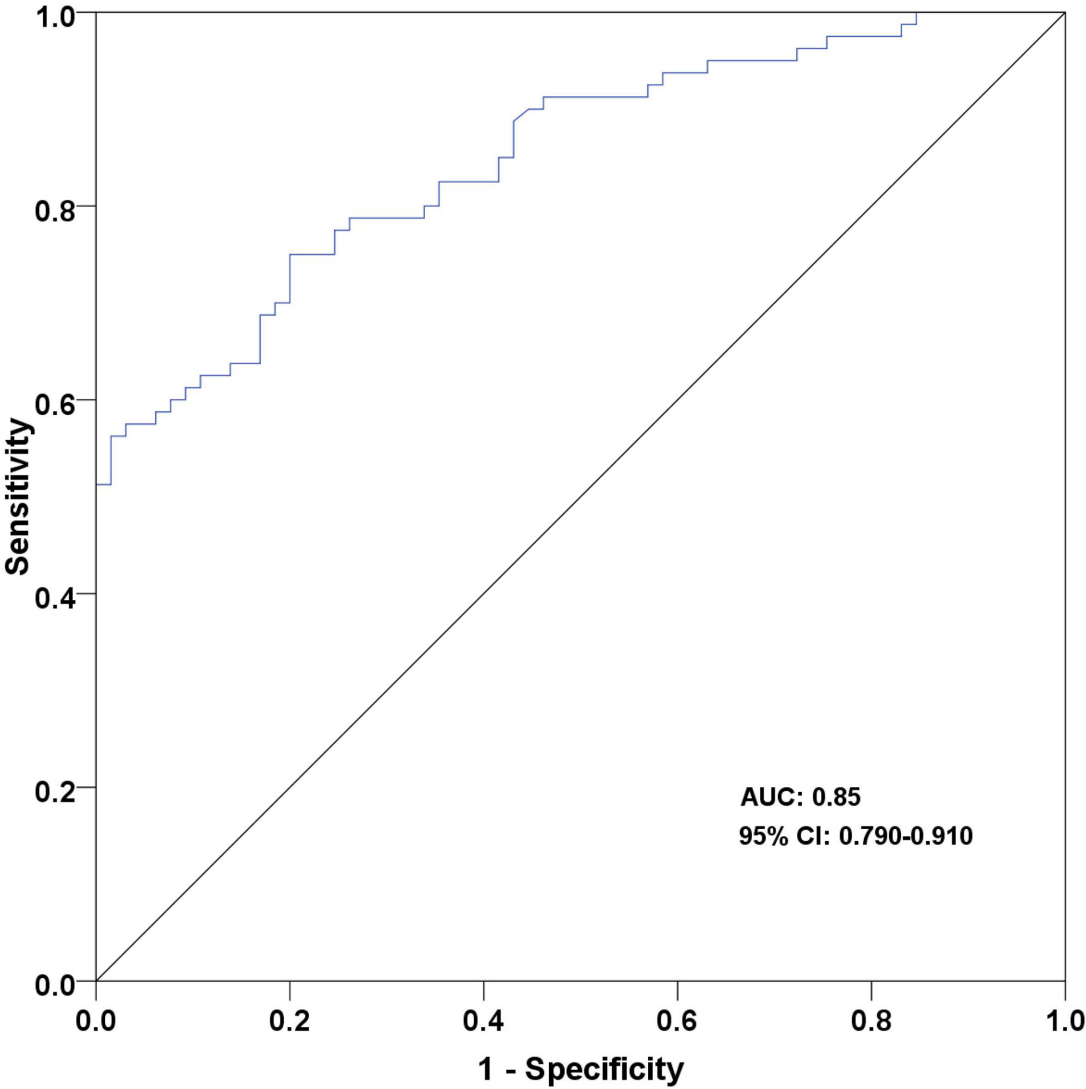
The ROC curve of serum CgA levels. The sensitivity and specificity were 78.8% and 73.8% at the cutoff level of 46.2 ng/mL. The area under the curve (AUC) was 0.85 with 95% confidence interval of 0.79–0.91.

Characteristics and serum CgA levels of healthy individuals were shown in S2 Table. The median CgA level in patients with GEP-NETs prior to treatment (93.8 ng/mL; range: 21.7–2795.1 ng/mL) was found to be significantly higher than healthy individuals (37.1 ng/mL; range: 14.3–87.1 ng/mL; P<0.01; Fig 2). The cutoff value of CgA was defined as 46.2 ng/mL based on ROC analysis (sensitivity: 78.8%, specificity: 73.8%, AUC: 0.85; Fig 3).

### Correlation of CgA level to characteristics of patients

Levels of CgA were found to be significantly higher in patients with carcinoid syndrome (298.8 ng/mL; range: 52.0–913.4 ng/mL) than in patients without carcinoid syndrome (82.9 ng/mL; range: 21.7–2795.1 ng/mL; P = 0.011). Additionally, significantly higher CgA values were found in patients with liver metastasis (137.0 ng/mL; range: 21.7–2795.1 ng/mL) compared to patients without liver metastasis (64.4 ng/mL; range: 27.5–870.0 ng/mL; P = 0.023). There were no other significant differences found between levels of CgA in serum and other characteristics including age, gender, location, stage and grade (Table 1).

### Relationship between CgA level changes and clinical response

**Fig 4:**
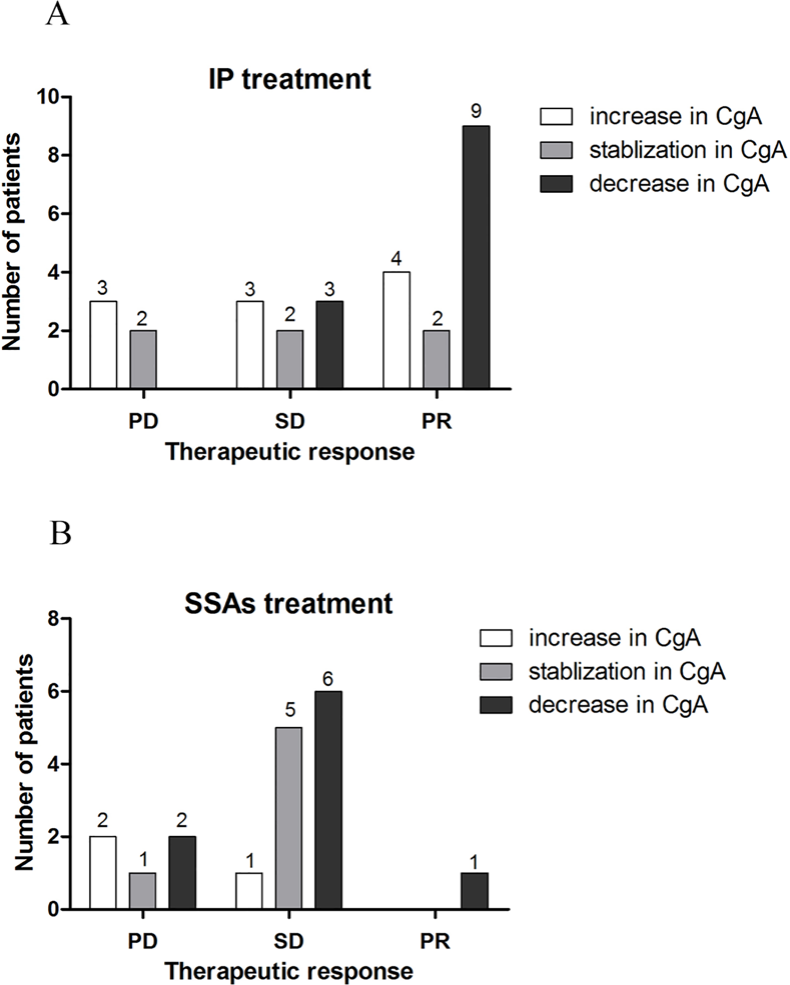
Relationship between CgA level changes and clinical response. (A) Of the twenty-eight patients that received IP chemotherapy as the first-line treatment, sixteen of twenty-three with non-progressive disease (PR and SD) showed decreased or stable CgA levels; three out of five with progressive disease (PD) exhibited increased CgA levels. (B) Of the eighteen patients who received somatostatin analogs as the first-line treatment, twelve of thirteen with non-progressive disease had decreased or stable CgA levels; two of the five patients having progressive disease showed increased CgA levels.

The detailed data of CgA level changes after surgery or 6 cycles of systemic treatment (IP regimen or SSAs) were shown in S3 and S4 Tables. During the course of this study, three patients underwent palliative surgery and CgA levels were found to be significantly decreased as a result. Among the patients that received IP chemotherapy, sixteen of twenty-three (69.6%) had non-progressive disease (PR and SD) where CgA levels decreased or remained stable following treatment; three out of five (60%) with progressive disease (PD) exhibited increased CgA levels following treatment (Fig 4). Spearman correlation test showed that a weak and non-significant association was observed between changes in CgA levels and clinical response to the IP regimen (r = 0.236, P = 0.226).

Of the twenty-two patients who received somatostatin analogs as the first-line treatment, there were eighteen that underwent CT scans and concomitant serum CgA examination following six cycles of treatment. Of the thirteen patients with non-progressive disease, twelve (92.3%) were found to have decreased or stable CgA levels. Of the five patients classified as having progressive disease, two of them (40%) showed increased CgA levels while three had decreased or stable CgA levels (Fig 4). A non-significant correlation was observed between changes in CgA levels and clinical response to SSAs (r = 0.388, P = 0.111).

**Fig 5:**
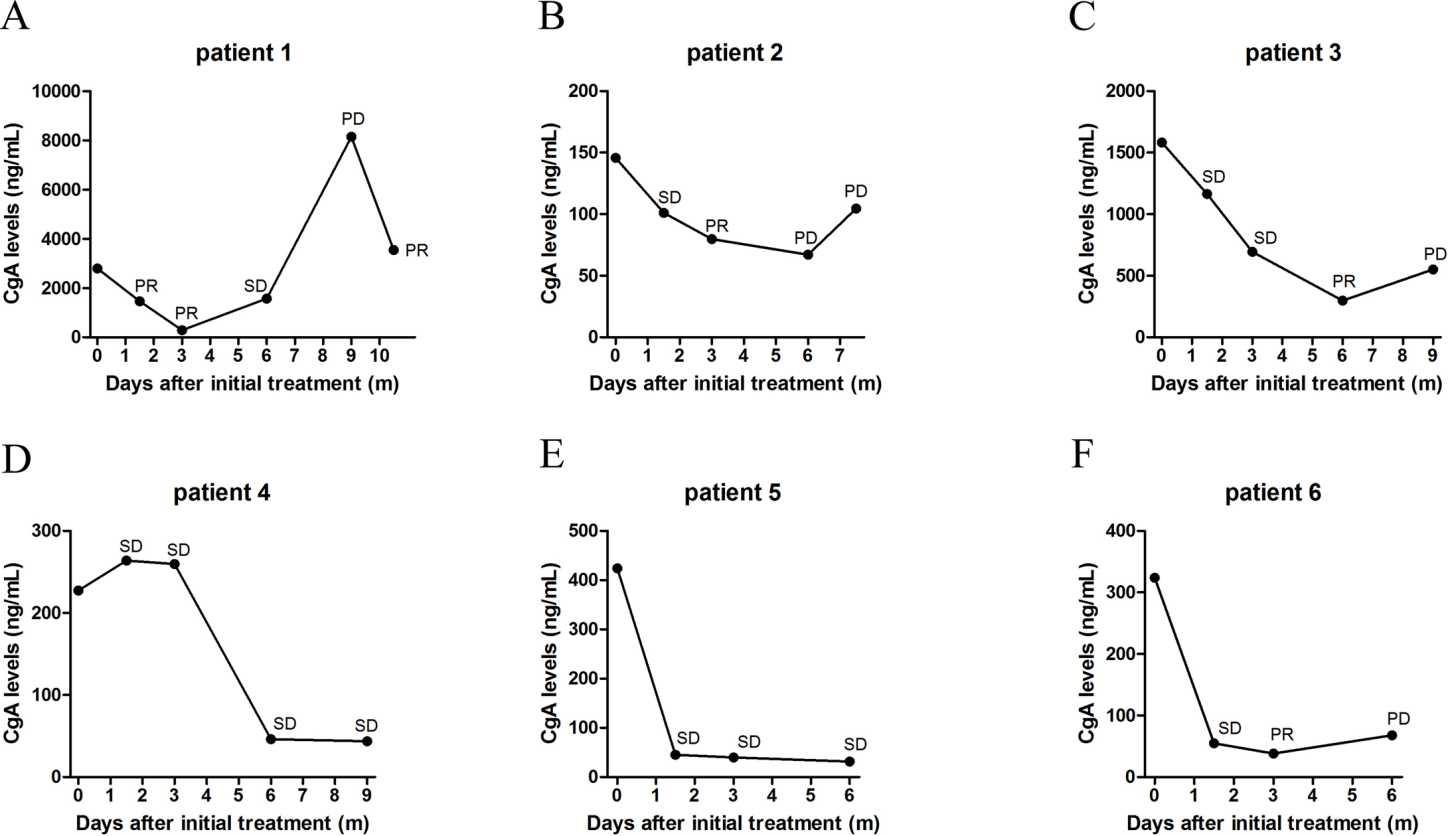
Dynamic changes of CgA levels during treatment. (A), Patient 1 was treated with IP regimen for three months (6 cycles) with partial response (PR) followed by 6 months (6 cycles) of somatostatin analogues with progressive disease (PD) and 3 cycles of IP regimen with PR. (B), Patient 2 was treated with 6 cycles of IP regimen with PR followed by 3 cycles of SSAs with PD and 3 cycles of IP regimen with PD. Patient 3 (C) and patient 4 (D) were treated with 6 cycles of IP regimen with SD followed by 6 cycles of SSAs. Patient 5 (E) and patient 6 (F) were treated with 6 cycles of IP regimen followed by 6 cycles of SSAs.

Serial measurements of CgA levels were taken from six patients treated with IP regimen chemotherapy followed by SSAs as maintenance treatment. Patients were evaluated for clinical response every three cycles of therapy. As shown in S5 Table, the concordance rate between clinical response and CgA level changes in the six patients were up to 95.7%. The data indicates that changes of CgA levels during treatment could be used as a helper method to dynamically monitor therapeutic response, though there is more research required in order to validate such claims (Fig 5).

### Correlation of CgA level to survival

**Fig 6:**
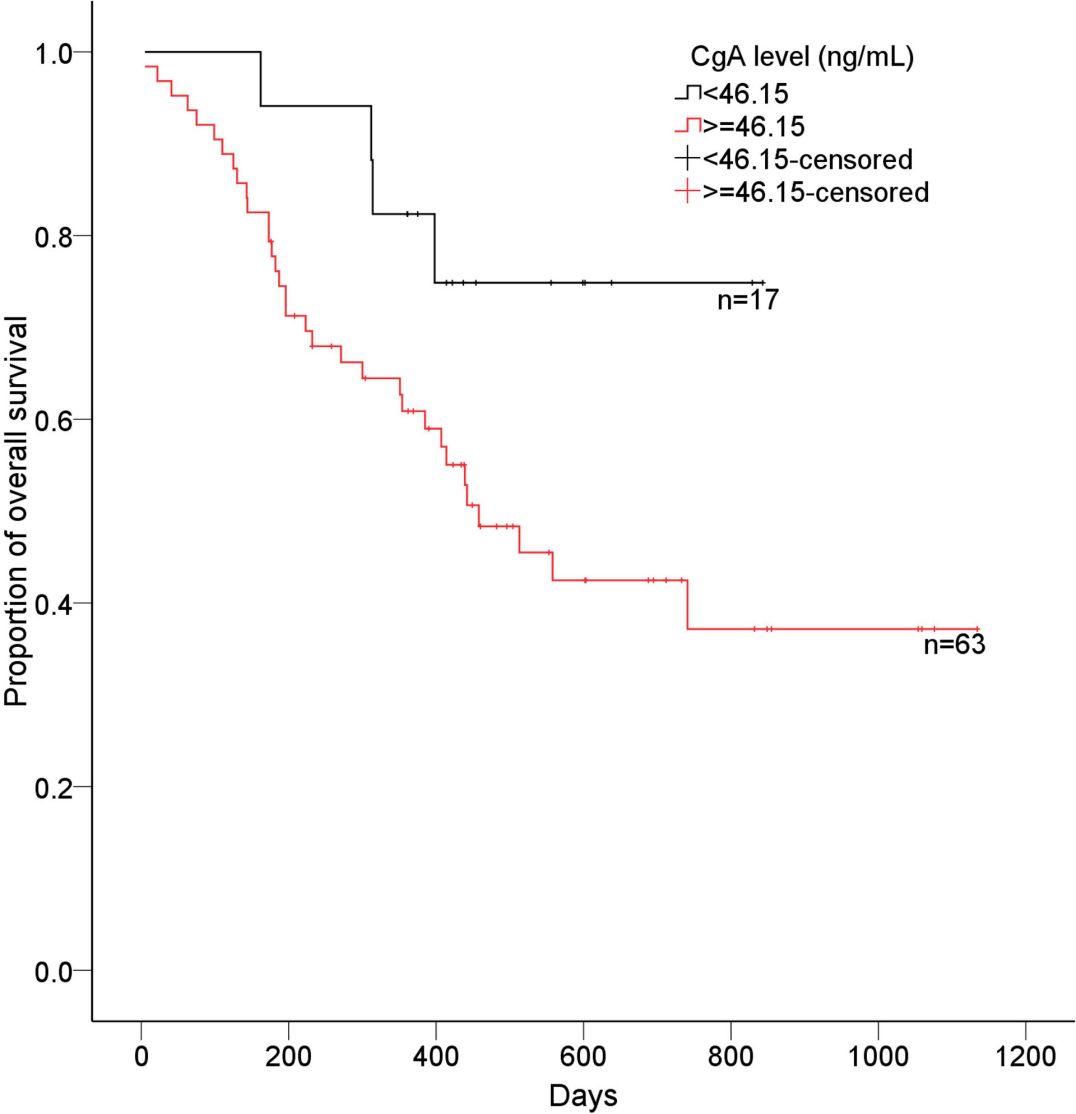
Overall survival curve of patients based on CgA levels. Patients with high CgA levels (≥46.2 ng/mL) had a significantly poorer survival than patients with low CgA levels (<46.2 ng/mL).

Up to October 2014, 79 of 80 patients were available for follow-up, and the median follow-up was 402 days (202 days-557 days). Of the 79 patients with follow-up, 38 had died. According to ROC curve, CgA level of 46.2 ng/mL was defined as cutoff value, therefore, patients with high CgA levels (CgA level ≥46.2 ng/mL; median overall survival: 392.5 days, 95% CI: 335.9–479.0) had a significantly poorer survival than patients with low CgA levels (CgA level <46.2 ng/mL; median overall survival: 437.5 days, 95% CI: 391.1–726.9; P = 0.045, Fig 6).

### Superiority of CgA compared to other blood markers

To compare the sensitivity of CgA with that of other blood markers for GEP-NETs, the levels of NSE, CEA and CA19-9 in these GEP-NETs patients were also tested (S6 Table). The sensitivity of serum CgA was not significantly different from that of NSE (78.8% vs 74.3%, P = 0.683) but was significantly greater than that of CEA (78.8% vs 22.4%, P<0.001) and CA19-9 (78.8% vs 17.3%, P<0.001). Sensitivity of NSE in the present study was higher than that in previous reports [4, 5], which may be due in part to the advanced tumor stage of patients in the study. For 11 patients, the NSE was found to be elevated though CgA levels were normal, which may indicate that combination of CgA and NSE could be used in clinical practice.

### Correlation between tissue and serum CgA expression

We retrospectively collected the immunohistochemical staining data of tissue CgA expression of the 80 patients. Collected data revealed that CgA expression in tumor tissue was available in 78 patients with 35 negative and 43 positive expression (a sensitivity of 55.1%). The phi-coefficient correlation test showed that tissue CgA expression was correlated with serum CgA levels (Phi coefficient = 0.308, P = 0.007) (S7 Table).

## Discussion

Accepted as the best characterized circulating biomarker in both the diagnosis of and follow up of GEP-NETs patients, CgA is currently not recommended for patients in China due in large part to the lack of systematic research on its clinical usefulness. Thus patients of Chinese descent diagnosed with advanced GEP-NETs, which comprise a majority of the diagnosed GEP-NETs, were evaluated for circulating levels of CgA. Results indicated that serum level of CgA at 46.2 ng/mL could reliably discriminate advanced GEP-NETs patients from healthy individuals with a high degree of sensitivity and specificity. Thus, it is plausible that serum CgA could be used as a reliable diagnostic biomarker of advanced GEP-NETs for patients of Chinese descent.

Well reviewed by Singh *et al*, there have been different cut off values of CgA used in previous studies to distinguish between healthy subjects and those with GEP-NETs [17]. From 95 ng/mL, reported by Wang *et* al, to a plasma value of 94 U/L utilized by Chou *et al* the level has been fairly open to discrimination based in part on several factors [9, 18]. Different patient populations, varied methods of detection, as well as sample types (serum versus plasma) [19] are all contributing factors which affect the cut-off point determined by the researcher. Further confounding the issue, it had been reported previously that levels of CgA varied with local or diffuse disease as well as the overall tumor burden [20, 21]. Important to the present study, which may have exerted an inordinate amount of influence, was the fact that all patients enrolled had advanced disease (stage III/IV).

Therapeutic response monitoring is mainly based on morphological evaluation using CT and magnetic resonance imaging (MRI) according to the RECIST criteria. However, imaging evaluation of treatment response is expensive and time-consuming. More importantly, RECIST classification cannot evaluate changes in non-target lesions. One important aim of this study was to evaluate the potential of using circulating CgA in predicting response to treatment in patients with advanced GEP-NETs. Despite the increased prevalence of GEP-NETs, the treatment of advanced GEP-NETs has evolved slowly. More recently, we evaluated the effect of IP regimen for high-grade GEP-NETs, also called gastroenteropancreatic neuroendocrine carcinomas (GEP-NECs), in our center and found that the overall response rate to IP regimen was 57.1%, indicating that IP chemotherapy is moderately effective in patients with GEP-NECs. In the present study, we further investigated whether changes in serum CgA levels is correlated with clinical response to chemotherapy with IP. The concordance rate between serum CgA level changes and clinical response to the IP regimen was 67.9% (19/28), while a non-significant and weak association was observed between them. Thus far, it appears as though this study is the first to explore the role of serum CgA in predicting therapeutic response to IP chemotherapy.

Patients with GEP-NETs and hormone-related symptoms or patients without hormone-related symptoms having a positive Octreoscan may be considered for treatment with SSAs [22]. In the present study, the correlation of CgA level changes and clinical response to six cycles of SSAs was investigated in 18 patients where it was found that the concordance rate between serum CgA level changes and clinical response to SSAs was 77.8% (14/18) and changes in serum CgA levels were not significantly correlated with response to SSAs.

There was a lack of association between changes in CgA levels and therapeutic responses in the present study. One possible explanation is that the sample size was small. Another possible explanation is that CgA levels were not only affected by tumor burden, they may also be affected by the tumor secreting activity [23]. A third explanation is that pre-existing conditions such as chronic atrophic gastritis, use of protein proton inhibitors as well as impaired kidney function may affect CgA levels [24, 25]. The results may be an indicator that CgA measurement alone may not adequately reflect tumor response to therapy. However, in cases where it is difficult to evaluate the disease by imaging methods alone such as immeasurable lesions, serum CgA measurement may be used as an auxiliary means of monitoring treatment response.

Multiple studies have previously reported an association between CgA levels and prognosis with controversial conclusions [11, 26–28]. In several studies, CgA levels were shown to be independent predictors of overall survival (OS) and progression-free survival (PFS) in patients with GEP-NETs [11, 26]. Conversely, Clancy *et al*. showed that increasing CgA levels were not independent predictors of decreased OS [29]. From this study it was found that the overall survival was shorter in patients with a high baseline of CgA compared to patients with low levels of CgA, indicating a potential role of CgA in prognosis evaluation. However, whether baseline CgA was an independent predictor of decreased overall survival in the multivariate analysis was not known. Interestingly, recent study has suggested that an early CgA response could be a better prognostic predictor compared with baseline CgA levels [30]. Further analysis is required in order to make a definitive statement regarding the use of circulating CgA as an indicator of prognosis.

A potential weakness of this study is that the number of patients used for evaluating whether changes in CgA levels were correlated with tumor response was relatively small. Further confounding the collection of data is the possibility that preexisting medical conditions could have yielded false positives [24, 25]. Further multi-center investigations with a larger number of patients are needed to validate the usefulness of CgA in the monitoring of patients with GEP-NETs and its prognostic role.

In summary, our study evaluated the circulating CgA level of advanced GEP-NETs in Chinese patients and demonstrated the potential role of circulating CgA level as a prognostic factor. More research are needed to validate the role of circulating CgA as a marker for monitoring clinical response in advanced GEP-NETs.

## Supporting Information

S1 File STROBE checklist v4 combined. (DOC)

S2 File PLOSOne Clinical Studies Checklist. (DOCX)

S3 File The Ethics Approval. (TIF)

S4 File The Study Protocol. (DOCX)

S1 Table Histopathological features of patients. (DOCX)

S2 Table Characteristics and serum CgA levels of healthy individuals. (DOCX)

S3 Table Changes in CgA levels and clinical response pre- and post-treatment with surgery and IP regimen. (DOCX)

S4 Table Changes in CgA levels and clinical response pre- and post-treatment with SSAs. (DOCX)

S5 Table Serial CgA level changes and clinical response in six patients. (DOCX)

S6 Table Expression of serum CgA and other blood markers. (DOCX)

S7 Table Correlation between tissue and serum CgA expression. (DOCX)
