# Supplementary figures and images for: Circulating Chromogranin A as A Marker for Monitoring Clinical Response in Advanced Gastroenteropancreatic Neuroendocrine Tumors
Source: PLoS One. 2016 May 9;11(5):e0154679. doi: 10.1371/journal.pone.0154679 (PMC4861261; doi:10.1371/journal.pone.0154679)

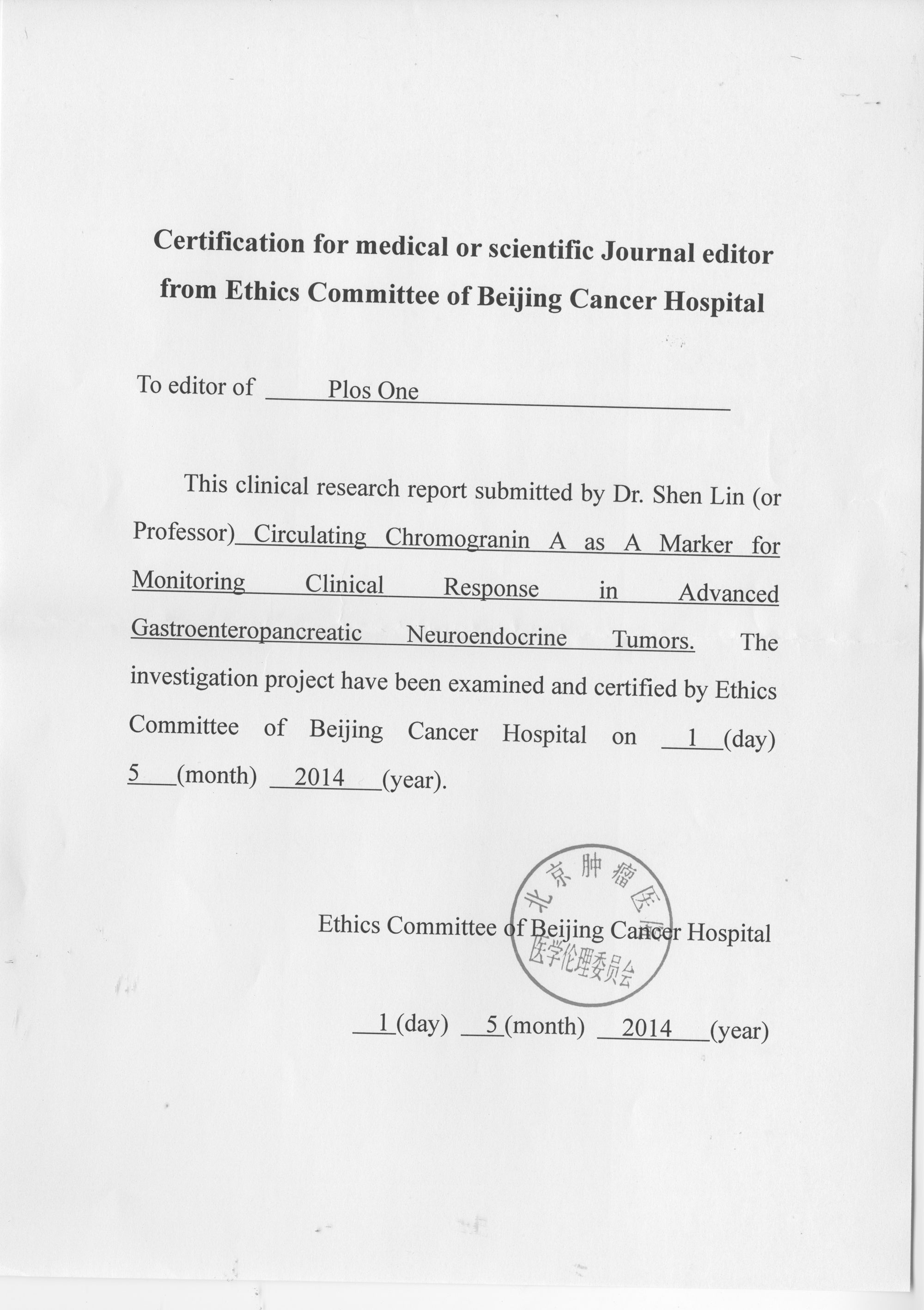

Supplement: S3 File — (TIF) [file pone.0154679.s003.tif]
