# Supplementary material for: Circulating Chromogranin A as A Marker for Monitoring Clinical Response in Advanced Gastroenteropancreatic Neuroendocrine Tumors
Source: PLoS One. 2016 May 9;11(5):e0154679. doi: 10.1371/journal.pone.0154679 (PMC4861261; doi:10.1371/journal.pone.0154679)
Supplement: S4 File — (DOCX) [file pone.0154679.s004.docx]

**Retrospective analysis of circulating chromogranin A in Advanced Gastroenteropancreatic Neuroendocrine Tumors**

**Study protocol**

| **Sponsor** | **Peking University Cancer Hospital & Institute** |
| --- | --- |
| **Investigators** | **Lin Shen MD.PHD and Jie Li MD.PHD** |
| **Version Number** | **Version 1.0** |
| **Version Date** | **2014.04.24** |

**The study protocol**

| Official scientific title | Retrospective analysis of circulating chromogranin A in Advanced Gastroenteropancreatic Neuroendocrine Tumors |
| --- | --- |
| Sponsor | Peking University Cancer Hospital & Institute |
| Investigators | Lin Shen MD.PHD  Jie Li MD.PHD |
| Brief summary | Chromogranin A (CgA) is the best characterized circulating biomarker in both the diagnosis of and follow up of GEP-NETs patients. However, CgA is currently not recommended for patients in China due in large part to the lack of systematic research on its clinical usefulness. The aim of the study is to study the diagnostic role as well as the prognostic role of circulating CgA in patients with GEP-NETs. The second purpose was to investigate whether circulating CgA is useful in evaluating clinical response in patients with advanced GEP-NETs. |
| Study type | Observational |
| Study design | Time perspective: retrospective |
| Sampling method | Non-probability sample |
| Study population | Patients with Advanced Gastroenteropancreatic Neuroendocrine Tumors |
| Condition | Advanced Gastroenteropancreatic Neuroendocrine Tumors |
| Intervention | Laboratory biomarker analysis  Retrospective data collection |
| Eligibility criteria | Inclusion criteria:   - Adult patients >=18 years of age - Inoperable locally advanced, recurrent, and/or metastatic Gastroenteropancreatic Neuroendocrine Tumors   Exclusion criteria:   - Previous therapy for advanced/metastatic disease - Blood samples were not available |
| Gender | Both |
| Ages | 18 years and older |
| Accepts healthy volunteers | Yes |
| Anticipate start date | May 2014 |
| Estimated enrollment | 80 |
| Estimated completion date | February 2015 |
| Contacts | Lin Shen; 010-88196088; lin100@medmail.com.cn |
