# Supplementary material for: Circulating Chromogranin A as A Marker for Monitoring Clinical Response in Advanced Gastroenteropancreatic Neuroendocrine Tumors
Source: PLoS One. 2016 May 9;11(5):e0154679. doi: 10.1371/journal.pone.0154679 (PMC4861261; doi:10.1371/journal.pone.0154679)
Supplement: S1 Table — (DOCX) [file pone.0154679.s005.docx]

**S1 Table. Histopathological features of patients.**

| **Histopathological features** | **No. of patients (%)** |
| --- | --- |
| **Ki-67 index** |  |
| 〈3% | 5 (6.3%) |
| 3-20% | 22(27.5%) |
| 〉20% | 53 (66.2%) |
| **Syn** |  |
| Positive | 72 (90%) |
| negative | 8 (10%) |
| **CD56** |  |
| Positive | 52 (74.3%) |
| negative | 18 (25.7%) |
