# Supplementary material for: Circulating Chromogranin A as A Marker for Monitoring Clinical Response in Advanced Gastroenteropancreatic Neuroendocrine Tumors
Source: PLoS One. 2016 May 9;11(5):e0154679. doi: 10.1371/journal.pone.0154679 (PMC4861261; doi:10.1371/journal.pone.0154679)
Supplement: S2 Table — (DOCX) [file pone.0154679.s006.docx]

**S2 Table. Characteristics and serum CgA levels of healthy individuals.**

| **Characteristics** | **No. of patients (%)** | **Median CgA level (ng/mL)** | **P value*** |
| --- | --- | --- | --- |
| **Gender** |  |  | 0.256 |
| Female | 35 (53.8%) | 34.3 (16.3-87.1) |  |
| Male | 30 (46.2%) | 32.9 (14.3-78.8) |  |
| **Age** |  |  | 0.243 |
| ≤57 | 38 (58.5%) | 36.1 (15.5-87.1) |  |
| >57 | 27 (41.5%) | 37.1 (14.3-81.3) |  |

***** The Mann–Whitney test was used.
