# Supplementary material for: Circulating Chromogranin A as A Marker for Monitoring Clinical Response in Advanced Gastroenteropancreatic Neuroendocrine Tumors
Source: PLoS One. 2016 May 9;11(5):e0154679. doi: 10.1371/journal.pone.0154679 (PMC4861261; doi:10.1371/journal.pone.0154679)
Supplement: S3 Table — (DOCX) [file pone.0154679.s007.docx]

**S3 Table. Changes in CgA levels and clinical response pre- and post-treatment with surgery and IP regimen.**

|  | **CgA levels (ng/mL)** | |
| --- | --- | --- |
| **No.** | **Before surgery** | **After surgery** |
| 1 | 150.3 | 24.8 |
| 2 | 212.6 | 34.8 |
| 3 | 38.3 | 26.7 |
| **No.** | **Prior treatment** | **PR of after treatment** |
| 1 | 58.7 | 25.7 |
| 2 | 2795.1 | 286.7 |
| 3 | 145.8 | 79.8 |
| 4 | 82.9 | 50.2 |
| 5 | 97 | 55.3 |
| 6 | 73.3 | 33.4 |
| 7 | 323.8 | 38 |
| 8 | 455.3 | 328.2 |
| 9 | 193.6 | 45.3 |
| 10 | 55.7 | 45 |
| 11 | 125.3 | 48.4 |
| 12 | 48 | 119.7 |
| 13 | 298.8 | 868 |
| 14 | 30 | 43.4 |
| 15 | 21.7 | 32.4 |
| **No.** | **Prior treatment** | **SD of after treatment** |
| 1 | 1078.9 | 2060.3 |
| 2 | 227.4 | 259.5 |
| 3 | 38.6 | 62.3 |
| 4 | 797.5 | 1495.3 |
| 5 | 67.1 | 71.7 |
| 6 | 720.5 | 106.9 |
| 7 | 1582.6 | 694.3 |
| 8 | 424 | 45.3 |
| **No.** | **Prior treatment** | **PD of after treatment** |
| 1 | 1742.8 | 3714.3 |
| 2 | 27.5 | 45.2 |
| 3 | 283.7 | 1113.3 |
| 4 | 38.1 | 41 |
| 5 | 233 | 185.1 |
