# Supplementary material for: Circulating Chromogranin A as A Marker for Monitoring Clinical Response in Advanced Gastroenteropancreatic Neuroendocrine Tumors
Source: PLoS One. 2016 May 9;11(5):e0154679. doi: 10.1371/journal.pone.0154679 (PMC4861261; doi:10.1371/journal.pone.0154679)
Supplement: S4 Table — (DOCX) [file pone.0154679.s008.docx]

**S4 Table. Changes in CgA levels and clinical response pre- and post-treatment with SSAs.**

|  | **CgA levels (ng/mL)** | |
| --- | --- | --- |
| **No.** | **Prior treatment** | **PR of after treatment** |
| 1 | 427.4 | 60.8 |
| **No.** | **Prior treatment** | **SD of after treatment** |
| 1 | 59.9 | 38.2 |
| 2 | 425.8 | 39.4 |
| 3 | 1115.8 | 118.6 |
| 4 | 39.7 | 23.3 |
| 5 | 110.6 | 43.9 |
| 6 | 2072.0 | 620.3 |
| 7 | 123.1 | 134.0 |
| 8 | 51.9 | 53.6 |
| 9 | 1690.5 | 1949.9 |
| 10 | 43.3 | 34.4 |
| 11 | 50.3 | 42 |
| 12 | 42.9 | 67.9 |
| **No.** | **Prior treatment** | **PD of after treatment** |
| 1 | 913.4 | 378.6 |
| 2 | 40.8 | 21.8 |
| 3 | 46.4 | 35.4 |
| 4 | 100.0 | 146.4 |
| 5 | 38.7 | 62.0 |
