# Supplementary material for: Circulating Chromogranin A as A Marker for Monitoring Clinical Response in Advanced Gastroenteropancreatic Neuroendocrine Tumors
Source: PLoS One. 2016 May 9;11(5):e0154679. doi: 10.1371/journal.pone.0154679 (PMC4861261; doi:10.1371/journal.pone.0154679)
Supplement: S5 Table — (DOCX) [file pone.0154679.s009.docx]

**S5 Table. Serial CgA level changes and clinical response in six patients.**

| **Patients** | **Clinical response** | **CgA level** | **CgA level changes** | **Concordance rate** |
| --- | --- | --- | --- | --- |
| **1** | initial | 2795.1 |  | 100% |
|  | PR | 1461 | ↓^*^ |  |
|  | PR | 286.7 | ↓ |  |
|  | SD | 1569.1 | ↓ |  |
|  | PD | 8143.3 | ↑ |  |
|  | PR | 3546.3 | ↓ |  |
|  | initial | 145.8 |  | 75% |
| **2** | SD | 101 | ↓ |  |
|  | PR | 79.8 | ↓ |  |
|  | PD | 67 | ↓ |  |
|  | PD | 104.5 | ↑ |  |
|  | initial | 1582.6 |  | 100% |
| **3** | SD | 1164 | ↓ |  |
|  | SD | 694.3 | ↓ |  |
|  | PR | 298.3 | ↓ |  |
|  | PD | 551.2 | ↑ |  |
|  | initial | 227.4 |  | 100% |
| **4** | SD | 263.9 | ─ |  |
|  | SD | 259.5 | ─ |  |
|  | SD | 46.1 | ↓ |  |
|  | SD | 43.7 | ↓ |  |
|  | initial | 424 |  | 100% |
| **5** | SD | 45.3 | ↓ |  |
|  | SD | 39.9 | ↓ |  |
|  | SD | 31.3 | ↓ |  |
|  | initial | 323.8 |  | 100% |
| **6** | SD | 54.7 | ↓ |  |
|  | PR | 38 | ↓ |  |
|  | PD | 67.8 | ↑ |  |
| **Total** |  |  |  | 95.7% |

*↓: decreased CgA levels; ↑: elevated CgA levels; ─: stable CgA levels
