# Supplementary material for: Circulating Chromogranin A as A Marker for Monitoring Clinical Response in Advanced Gastroenteropancreatic Neuroendocrine Tumors
Source: PLoS One. 2016 May 9;11(5):e0154679. doi: 10.1371/journal.pone.0154679 (PMC4861261; doi:10.1371/journal.pone.0154679)
Supplement: S6 Table — (DOCX) [file pone.0154679.s010.docx]

**S6 Table. Expression of serum CgA and other blood markers.**

| **Other blood marker** |  | **Serum CgA (n)** | | **P value*** |
| --- | --- | --- | --- | --- |
|  |  | **+** | **-** |  |
| **NSE (n=74)** | **+** | 44 | 11 | 0.683 |
|  | **-** | 13 | 6 |  |
| **CEA (n=76)** | **+** | 13 | 4 | <0.001 |
|  | **-** | 46 | 13 |  |
| **CA19-9 (n=75)** | **+** | 13 | 0 | <0.001 |
|  | **-** | 45 | 17 |  |

*The McNemar’s test was used.
