# Supplementary material for: Circulating Chromogranin A as A Marker for Monitoring Clinical Response in Advanced Gastroenteropancreatic Neuroendocrine Tumors
Source: PLoS One. 2016 May 9;11(5):e0154679. doi: 10.1371/journal.pone.0154679 (PMC4861261; doi:10.1371/journal.pone.0154679)
Supplement: S7 Table — (DOCX) [file pone.0154679.s011.docx]

**S7 Table. Correlation between tissue and serum CgA expression**

| **Serum CgA** | **Tissue CgA** | | **total** | **P value*** |
| --- | --- | --- | --- | --- |
|  | **+** | **-** |  |  |
| **+** | 39 | 23 | 62 | 0.007 |
| **-** | 4 | 12 | 16 |  |
| **total** | 43 | 35 | 78 |  |

*The phi-coefficient correlation test was used.
